# Efficacy and mechanism of action of ginsenoside Rg3 on radiation proctitis in rats

**DOI:** 10.1002/iid3.70015

**Published:** 2024-09-24

**Authors:** Xuxia Li, Lili Lin, Xiaoyu Duan, Jiuju Dai, Tingting Hu, Hongyi Cai

**Affiliations:** ^1^ The First Clinical Medical College of Gansu University of Traditional Chinese Medicine Lanzhou China; ^2^ Department of Oncology Suqian First People's Hospital Suqian China; ^3^ Department of Radiotherapy Gansu Provincial Hospital Lanzhou China

**Keywords:** efficacy, ginsenoside Rg3, mechanism, radiation proctitis, TNF‐α/caspase‐8

## Abstract

**Objective:**

Radiation proctitis (RP) refers to rectal injury caused by radiation treatment of pelvic and retroperitoneal malignancies, which has a major impact on the treatment prognosis and quality of life of patients with cancer. The tetracyclic triterpene saponin monomer ginsenoside Rg3 (GRg3), the primary bioactive ingredient in ginseng extracts, has therapeutic effects against RP in rats. Here, we validated its efficacy and elucidated its mechanism of action.

**Methods:**

A rat RP model was established in 48 Wistar rats. Rats were randomly divided into control (untreated), irradiation, irradiation + dexamethasone, and irradiation + GRg3 (low‐, medium‐, and high‐dose) groups. After 2 weeks' treatment, serum IL‐4, IL‐10, and TNF‐α levels were tested by enzyme‐linked immunosorbent assays. In rectal tissue, *Ikbkb, Ikka*, and *Casp8* mRNA expression was detected by a reverse transcription‐quantitative polymerase chain reaction. IKK‐β, IκB‐α, p‐IκB‐α, p50, and caspase‐8 protein levels were determined by western blot analysis.

**Results:**

GRg3 significantly improved the general condition and histopathological damage in rats with RP. Moreover, GRg3 decreased the levels of factors that promote inflammation (TNF‐α) and increased the levels of factors that reduce inflammation (IL‐4 and IL‐10). GRg3 markedly reduced the activation of NF‐κB and caspase‐8 signaling pathways.

**Conclusions:**

Thus, GRg3 may reduce the inflammatory response by blocking the NF‐κB signaling pathway and improving the balance of inflammation‐related factors. GRg3 may also inhibit intestinal cell apoptosis by suppressing the TNF‐α/caspase‐8 signaling cascade, thereby reducing radiological rectal injury. Our results verify that GRg3 is a promising therapeutic agent for RP treatment and shed light on its mechanism.

## Graphical abstract

Study on the Efficacy and Mechanism of Action of Ginsenoside Rg3 on Rats with Radiation Proctitis.

## 1 INTRODUCTION

Radiotherapy is an important component of the multidisciplinary and comprehensive treatment model for pelvic and abdominal malignancies. Due to the fixed anatomical position of the rectum at the edge of the pelvis, which is close to the irradiated organs subjected to radiotherapy, rectal injury caused by ionizing radiation is one of the most common complications of pelvic and abdominal radiotherapy, which is also known as radiation proctitis (RP).^1^ Approximately 75% of all patients treated with pelvic radiotherapy suffer from acute RP (ARP) symptoms, including abdominal pain and diarrhea, urgency of bowel movements, and myxoid secretions. Although most ARP symptoms resolve after discontinuing radiotherapy, up to 20% of patients may develop chronic RP (CRP) or more severe outcomes such as intestinal fistula and hemorrhage, thereby requiring radiotherapy interruption, which seriously affects the efficacy of treatment and reduces the survival rate.^2^ Clinical treatment for RP is mostly aimed at resolving acute and chronic symptoms, but long‐term treatment can also produce side effects such as drug dependence and constipation;^3^ as a result, there is an urgent need to develop a new therapeutic avenue to address the etiology of RP.

**Figure 1:**
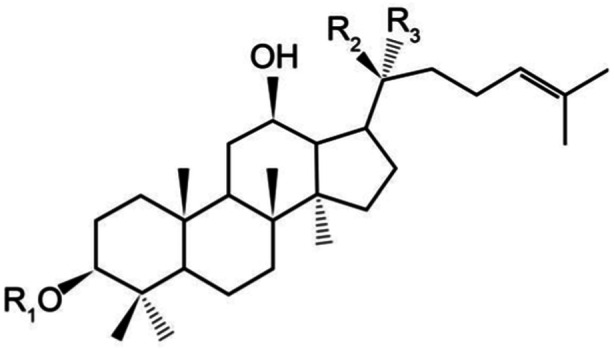
Chemical structure of Ginsenoside Rg3.

Ginsenoside Rg3 (GRg3), a tetracyclic triterpenoid saponin monomer, is the main bioactive component of ginseng extract, and its chemical structure is shown in Figure 1. It is commonly used clinically in the adjuvant treatment of tumor related diseases. In addition to directly exerting antitumor effects, GRg3 can also improve the sensitivity of tumors to chemotherapy and radiotherapy.^4^, ^5^, ^6^ Previous study showed that GRg3 has a therapeutic effect on radiation‐induced RP in rats.^7^ This study aimed to further validate its efficacy and mechanism that will hopefully provide a theoretical basis for future clinical applications.

## 2 SUBJECTS AND METHODS

### 2.1 Materials

Shenyi (GRg3) capsules were purchased from Jilin Yatai Pharmaceutical Company, Ltd. Sodium pentobarbital was purchased from Sinopharm Chemical Reagent Co., Ltd., and 0.9% saline and dexamethasone sodium phosphate injections were provided by the Radiotherapy Department of Gansu Provincial Hospital. Anti‐caspase‐8, ‐β‐actin, and ‐proliferating cell nuclear antigen (PCNA) antibodies were purchased from Boster Biological Technology Co., Ltd. Anti‐IκB‐α, ‐IKK‐β, and ‐NF‐κB p50 antibodies were purchased from Proteintech Group. The anti‐phosphorylated (p)‐IκB‐α antibody was purchased from Signalway Antibody LLC. The enzyme standards were purchased from Thermo Fisher Scientific. The linear gas pedals were purchased from Siemens.

### 2.2 Experimental model

Forty‐eight male Wistar rats (license number: SYXK [Gan] 2013‐0003), weighing approximately 180–200 mg, were acclimatized in the experimental animal room of the Gansu Provincial People's Hospital for 7 days before experiments commenced. The ambient temperature was maintained at 21–27°C, relative humidity at 50%–60%, and airflow velocity at 20 cm^2^/s, with air changes occurring 15 times/min. The rats were fed a maintenance diet, allowed to drink freely, and subjected to 12 h of alternating light and dark conditions. The experiments were conducted in compliance with the rules and regulations of the Medical Ethics Committee of Gansu Provincial Hospital (2022‐429, approved on January 11, 2023). All sections of this report adhere to the ARRIVE Guidelines for reporting animal research.^8^ A completed ARRIVE guidelines checklist is included in supplementary materials (Checklist S1).

The rats were randomly divided into six groups: control, irradiation (IR), irradiation + dexamethasone treatment (IR + DEX), and irradiation + low‐dose GRg3‐treatment (IR+low‐dose GRg3), medium‐dose GRg3 treatment (IR+medium‐dose GRg3), and high‐dose GRg3 treatment (IR+high‐dose GRg3). Except for the control group, all groups were anesthetized with 3% pentobarbital sodium at a dose of 40 mg/kg after 12 h of fasting before irradiation. After anesthesia, the rats were fixed in a supine position on a wooden board after their limbs were completely relaxed. Irradiation was performed using 6 MV X‐rays at a dose rate of 300 cGy/min and an irradiation dose of 21.5 Gy. Rats were irradiated from the pubic symphysis to the anus, which was not surrounded by the lead block. After irradiation, the rats were released from fixation, kept warm to avoid hypothermic death, and transported to the experimental animal room for further feeding after they awakened. Successful modeling was based on the presence of decreased mental and dietary status, body weight, and changes in stool characteristics, such as black, mucus‐like, or loose stools.

### 2.3 Treatment

Treatments were administered to the rats by gavage at the same time every day for 14 days, starting from the 8th day after modeling. Dexamethasone concentrations were selected based on a previous study by Wang et al.,^9^ and GRg3 concentrations were derived from a previous work.^7^, ^10^ The control and IR groups were administered saline at 40 mL/kg per day; the IR + DEX group was administered dexamethasone solution at 1.425 mg/kg. For the GRg3 groups, a 4 mg/mL GRg3 suspension was prepared and administered to the low‐, medium‐, and high‐dose GRg3‐treatment groups at 20, 40, and 80 mg/kg, respectively.

### 2.4 Observation indices and tissue collection

The rats were observed for daily food and water intake, fecal changes, voluntary activity, and reaction speed, and were weighed and recorded at the same time each day. After 2 weeks' treatment, rats were anesthetized, and 5 mL of abdominal aortic blood was collected. Rats were then executed and dissected. Approximately 4 cm of rectal tissue was collected, 2 cm above the anus. The rectal tissue was split along the longitudinal axis, washed in saline, and excess water was absorbed using filter paper. One part of the tissue specimen was fixed with 4% paraformaldehyde solution for pathological section observation, and the other was transferred to a −80°C freezer with liquid nitrogen in lyophilization tubes as backup samples.

### 2.5 Enzyme‐linked immunosorbent assay (ELISA)

ELISAs were used to test the serum IL‐4, IL‐10, and TNF‐α levels in each group of rats. Rat IL‐4, IL‐10, and TNF‐α ELISA kits (Lanzhou Kebao Biological Company) were used according to the manufacturer's instructions. Analyses were performed using the DS‐11 ultra‐microspectrophotometer (DeNovix).

### 2.6 Reverse transcription‐quantitative polymerase chain reaction (RT‐qPCR)

Total RNA was extracted using TRIeasy^TM^ total RNA extraction kits (Lanzhou Kebao Biological Company). Reverse transcription kits were purchased from Lanzhou Kebao Biological Company, and the mRNA expression levels of *Ikbkb, Ikba*, and *Casp8* were detected by RT‐qPCR using the PTC‐200 reverse transcription PTC‐200 thermocycler (Bio‐Rad) and a fluorescence quantitative PCR instrument (Roche).

### 2.7 Western blot analysis

Cytoplasmic and nucleolar proteins were extracted using nucleoprotein and plasma protein extraction kits (Shenyang Wanlei Biotechnology Company) according to the manufacturer's instructions. IKK‐β, IκB‐α, p‐IκB‐α, p50, and caspase‐8 protein contents were determined by western blot analysis using fluorescence quantification kits (Lanzhou Kebao Biological Company) and a gel imager (Bio‐Rad).

### 2.8 Statistical analysis

SPSS software (version 24.0; IBM SPSS Inc.) was used for data analysis and processing. ImageJ software (NIH) was used for image analysis. GraphPad Prism 8 (GraphPad Inc.) was used to create the graphs. Quantitative data with a normal distribution were expressed as mean ± standard deviation. A one‐way analysis of variance was used to compare groups. As post hoc tests, the Bonferroni test was employed when the variance was equal, and Dunnett's T3 test was used when it was not. A *P*‐value of <0.05 was deemed statistically significant.

## 3 RESULTS

### 3.1 General condition

The rats in the control group were in good general condition, responsive, and had normal mental and activity statuses. Feeding and drinking gradually increased with body weight; the rats' fur was neat and lustrous, and their excrement was granular. On Days 3–5 after irradiation, experimental rats began to show different degrees of RP symptoms. Five rats had black stools, 16 had myxoid stools, eight had paste‐like stools, and five had loose stools. Three rats developed cachexia. Compared with the control group, the food and water intake and activity of rats in the experimental groups were significantly reduced. These rats preferred to huddle in corners. When grasped, the rats offered weak resistance. Their perianal fur was dirty and partially shed. One cachectic rat died on the 6th day after irradiation. On the 8th day, treatment by oral gavage was started. The rats in the control group ate as usual, whereas the rats in the IR group had decreased food and water intake and showed the most severe enteritis symptoms. After 8 days of saline gavage, the amount of food consumed in the IR group was approximately half of that before gavage treatment. The dexamethasone‐ and GRg3‐treated rats showed no significant changes in status during the first 3 days of treatment. From the 4th day, the rats' feeding and drinking gradually increased, and their feces gradually normalized. Compared with the IR group, after 2 weeks of gavage, rats in the high‐dose GRg3‐treatment group showed significantly improved mood and activity, and their hair became shiny. During the gavage treatment period, one rat in each of the IR and low‐dose GRg3‐treatment groups died.

### 3.2 Changes in weight

Before irradiation, body weight did not differ between the groups (*p* > 0.05). Six days postirradiation, the weight of rats in each experimental group was significantly lower than that in the control group (*p* < 0.05) but did not differ across the experimental groups (*p* > 0.05). After gavage treatment, the body weight of the control group rats gradually increased by 2–3 g per day, whereas that of rats in the IR group continuously decreased. The weight of the rats in the groups treated with GRg3 and dexamethasone showed no significant change during the first 3 days of treatment but gradually increased from the 4th day by 1–3 g per day. Compared with the IR group, the body weight of the rats in the dexamethasone‐treatment group and the high‐dose GRg3‐treatment group was significantly increased by the 8th and 14th days of treatment, and the difference was statistically significant (*p* < 0.05) (Table 1).

**Table 1:** Changes in weight of rats in each group at different timepoints (g, *x̄* ± *s*).

Group	Pre‐irradiation	6‐days postirradiation	2nd day of treatment	8th day of treatment	14th day of treatment
Control	188.92 ± 3.20	202.22 ± 3.74	208.73 ± 4.36	225.80 ± 3.63	238.88 ± 4.29
Model group	189.39 ± 5.87	179.70 ± 5.80*	173.63 ± 7.33*	164.99 ± 15.03*	171.36 ± 18.05*
Dexamethasone‐treatment group	186.00 ± 5.07	176.91 ± 5.20*	171.90 ± 4.58*	181.37 ± 4.57*,**	193.57 ± 5.22*,**
Low‐dose GRg3 group	184.98 ± 4.79	176.27 ± 5.63*	171.89 ± 7.63*	174.33 ± 11.95*	187.11 ± 11.22*
Medium‐dose GRg3 group	188.70 ± 5.93	179.14 ± 6.04*	174.99 ± 6.63*	178.35 ± 8.01*	191.12 ± 7.93*,**
High‐dose GRg3 group	184.77 ± 5.78	175.39 ± 6.26*	170.74 ± 6.47*	181.61 ± 7.33*,**	193.86 ± 7.56*,**

Abbreviation: GRg3, Ginsenoside Rg3.

*p < 0.05 versus blank control group; **p < 0.05 versus model group.

### 3.3 Histopathological changes in the rectum of rats in each group

**Figure 2:**
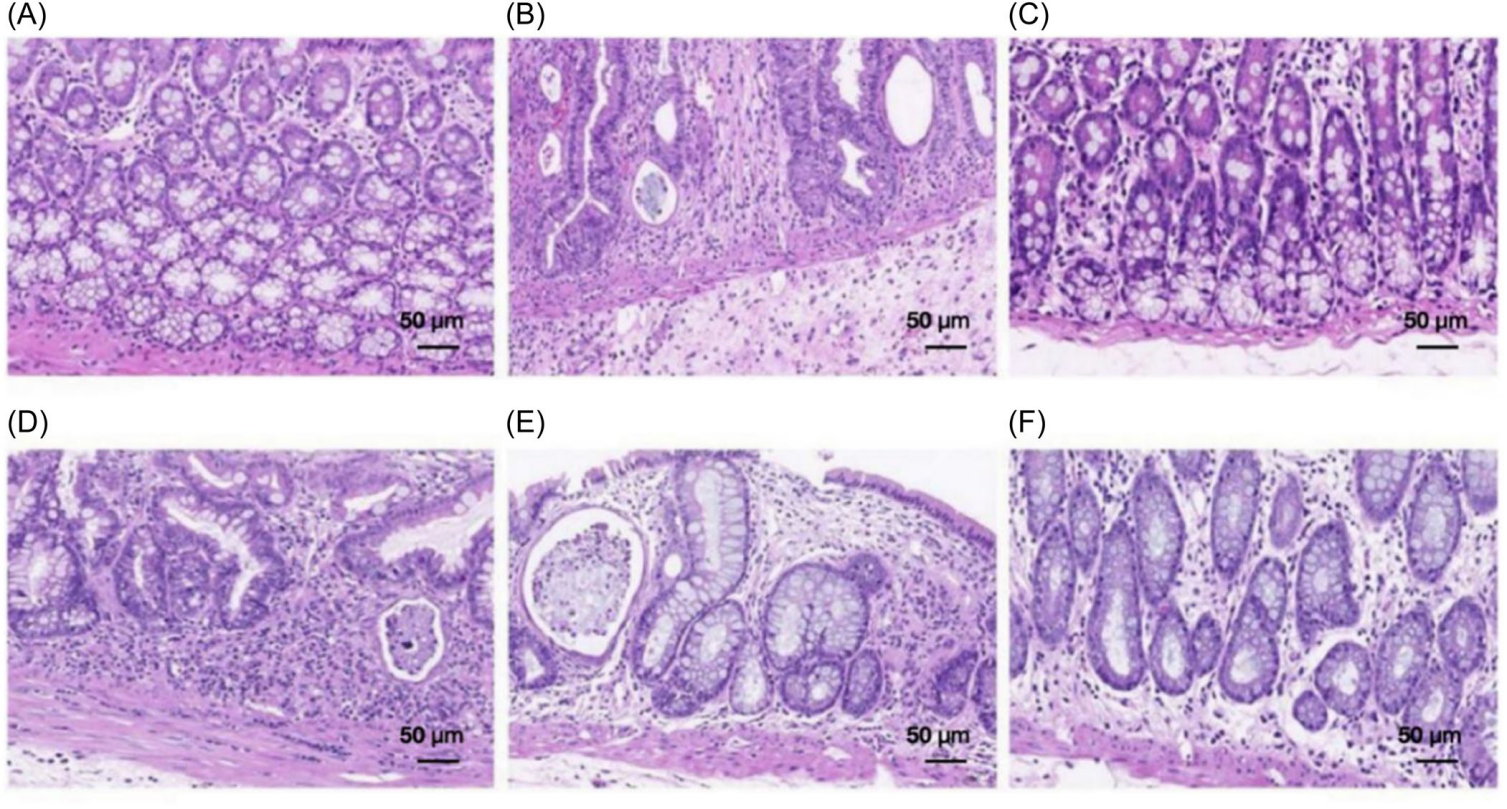
Histopathological results of the rat rectum (hematoxylin and eosin; × 200). (A) Blank control group. (B) Model group. (C) Dexamethasone‐treatment group. (D) Low‐dose GRg3‐treatment group. (E) Medium‐dose GRg3‐treatment group. (F) High‐dose GRg3‐treatment group. GRg3, Ginsenoside Rg3.

Histopathology of the rectal tissues is shown in Figure 2. In the control group, the rectal tissue structure was evident, the mucosal epithelium remained intact, the intestinal glands were numerous and closely spaced, and no obvious abnormalities were found in the mucosal layer. Compared with the control group, the rectal tissues in the IR group were extensively ulcerated, many intestinal glands had been replaced by hyperplastic connective tissue, some of the intestinal glands were dilated, and necrotic cell fragments were observed in the lumen. More lymphocytes were found in the mucosal layer, with severe submucosal edema and bleeding.

The rectal tissues of the dexamethasone‐treatment group showed slight inflammatory cell infiltration in the mucosal layer; otherwise, the tissues were structurally unaltered. In the low‐dose GRg3‐treatment group, the rectal tissues showed extensive ulceration, the shape and size of the intestinal glands were altered, the number was reduced, and some intestinal glands were dilated, with necrotic cell fragments visible in the lumen. The damage invaded the submucosa, and the mucosal, submucosal, and muscle layers showed significant infiltration of inflammatory cells. Pathological intestinal sections of mice in the middle‐dose GRg3‐treatment group showed a small number of necrotic and detached mucosal epithelial cells. The intestinal glands had different shapes and sizes, necrotic cell debris was observed in the gland cavity, and moderate inflammatory cell infiltration was observed around the intestinal glands. In the high‐dose GRg3 group, the rectal tissue structure was intact, the mucosal layer was edematous, and the intestinal glands were loosely arranged, with inflammatory cell infiltration.

### 3.4 Serum IL‐4, IL‐10, and TNF‐α levels of rats in each group

**Figure 3:**
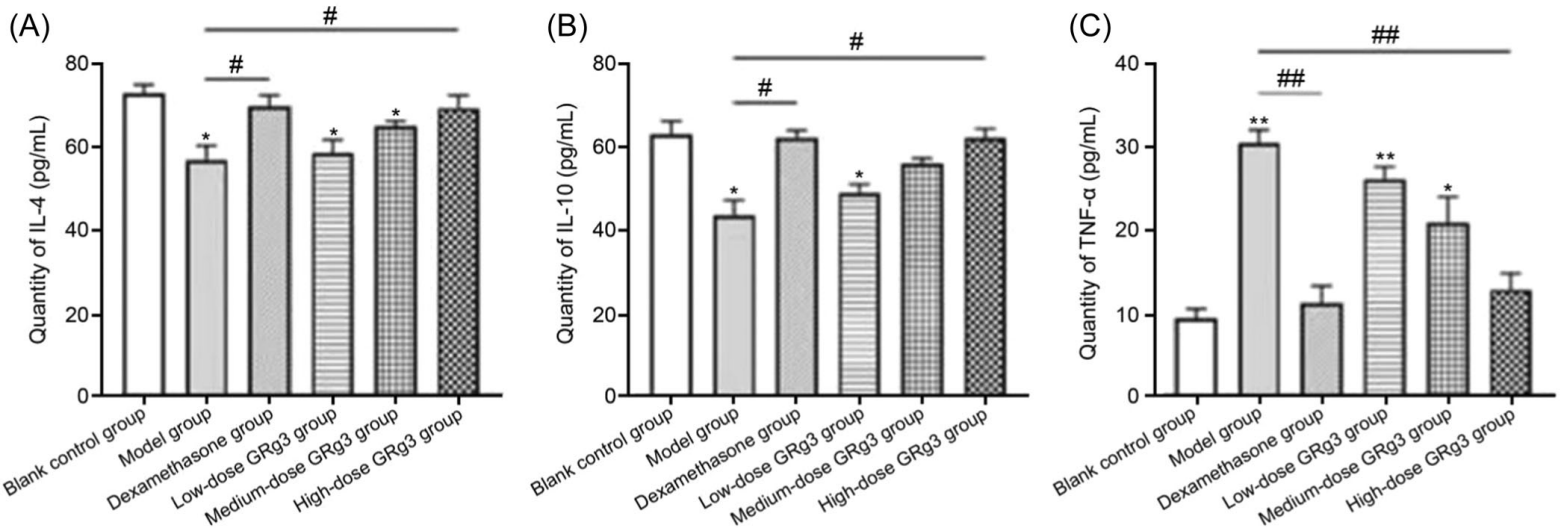
Serum IL‐4, IL‐10, and TNF‐α levels of rats in each group. (A) IL‐4. (B) IL‐10. (C) TNF‐α. 1: Blank control group. 2: Model group. 3: Dexamethasone‐treatment group. 4: Low‐dose GRg3‐treatment group. 5: Medium‐dose GRg3‐treatment group. 6: High‐dose GRg3‐treatment group. *: *p* < 0.05 versus blank control group. **: *p* < 0.01 versus blank control group. ^#^: *p* < 0.05 versus model group. ^##^: *p* < 0.01 versus model group. GRg3, Ginsenoside Rg3.

Serum TNF‐α levels were increased and IL‐4 and IL‐10 levels were decreased in the IR group compared with the control group (*p* < 0.05). Serum TNF‐α levels were decreased and those of IL‐4 and IL‐10 were increased in the dexamethasone‐treatment group and the high‐dose GRg3‐treatment group compared with the IR rats (*p* < 0.05) (Figure 3).

### 3.5 Changes in *Ikbkb*, *Ikba*, and *Casp8* mRNA expression in rats' rectal tissue

**Figure 4:**
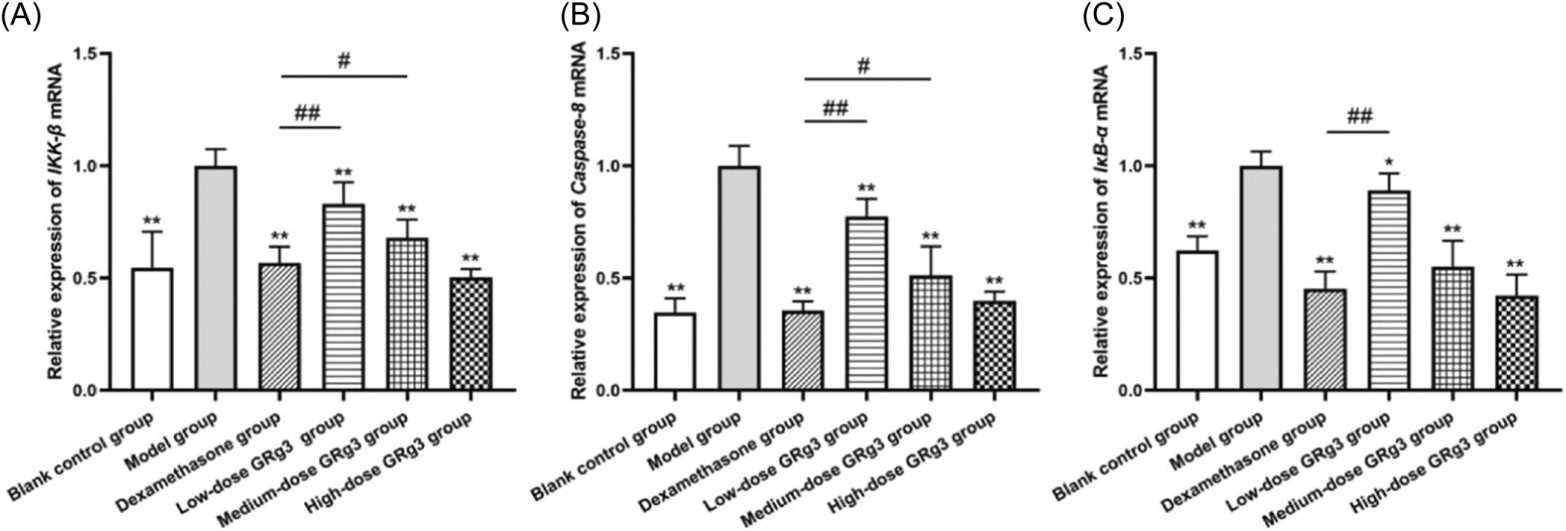
Relative expression levels of *Ikbkb*, *Ikba*, and *Casp8* mRNA in the rectal tissues of rats. (A) relative expression of *Ikbkb* mRNA. (B) relative expression of *Ikba* mRNA. (C) relative expression of *Casp8* mRNA. 1: Blank control group. 2: Model group. 3: Dexamethasone‐treatment group. 4: Low‐dose GRg3‐treatment group. 5: Medium‐dose GRg3‐treatment group. 6: High‐dose GRg3‐treatment group. *: *p* < 0.05 versus model group. **: *p* < 0.01 versus model group. ^#^: *p* < 0.05 versus dexamethasone‐treatment group. ^##^: *p* < 0.01 versus dexamethasone‐treatment group. GRg3, Ginsenoside Rg3.

RT‐qPCR results showed that the *Ikbkb*, *Ikba*, and *Casp8* mRNA expression levels in the rectal tissues of rats in the IR group were significantly higher than those in the control group (*p* < 0.01). After different interventions, the *Ikbkb*, *Ikba*, and *Casp8* mRNA levels in each treatment group decreased compared with the IR group (*p* < 0.05), and the expression of each of these factors tended to decrease with increasing GRg3 treatment doses. The differences in *Ikbkb*, *Ikba*, and *Casp8* mRNA expression between the dexamethasone‐treated group and the high‐dose GRg3‐treatment group were not statistically significant (*p* > 0.05) (Figure 4).

### 3.6 Changes in IKK‐β, IκB‐α, p‐IκB‐α, NF‐κB p50, and caspase‐8 protein levels in rats' rectal tissue

**Figure 5:**
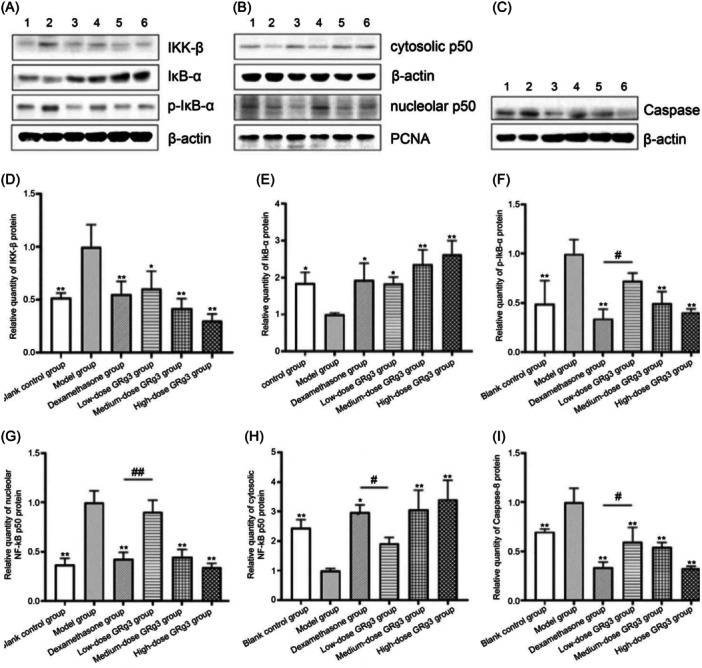
Relative levels of IKK‐β, IκB‐α, p‐IκB‐α, cytoplasmic p50, nucleolar p50, and caspase‐8 protein in rat rectal tissue. (A) The levels of IKK‐β, IκB‐α, and p‐IκB‐α proteins were detected by western blot analysis. (B) The levels of cytoplasmic p50 and nucleolar p50 proteins were detected by western blot analysis. (C) Caspase‐8 protein content was analyzed by western blot analysis. (D) IKK‐β. (E) IκB‐α. (F) p‐IκB‐α. (G) Nucleolar NF‐κB p50. (H) Cytoplasmic NF‐κB p50. (I) Caspase‐8. 1: Blank control group. 2: Model group. 3: Dexamethasone‐treatment group. 4: Low‐dose GRg3‐treatment group. 5: Medium‐dose GRg3‐treatment group. 6: High‐dose GRg3‐treatment group. *: *p* < .05 versus model group. **: *p* < 0.01 versus model group. ^#^: *p* < 0.05 versus dexamethasone‐treatment group. ^##^: *p* < 0.01 versus dexamethasone‐treatment group. GRg3, Ginsenoside Rg3.

Western blot analysis results showed that IKK‐β, p‐IκB‐α, nucleolar NF‐κB p50, and caspase‐8 protein levels were significantly higher in the rectal tissues of rats in the IR group than in those of the control group, whereas IκB‐α and nucleolar NF‐κB p50 protein levels were lower (*p* < 0.05). After treatment, IKK‐β, p‐IκB‐α, nucleolar NF‐κB p50, and caspase‐8 protein levels were decreased in the dexamethasone and medium‐ and high‐dose GRg3‐treatment groups compared with the IR group (*p* < 0.01), whereas IκB‐α and nucleolar NF‐κB p50 protein levels were significantly increased (*p* < 0.05). The protein levels of each factor were similar between the medium‐ and high‐dose GRg3‐treatment groups and the dexamethasone‐treatment group (*p* > 0.05) (Figure 5).

## 4 DISCUSSION

Herein, we validated the efficacy of GRg3, the primary bioactive ingredient in ginseng extracts, against RP in rats and elucidated its mechanism of action. We revealed that GRg3 reduces the inflammatory response via the NF‐κB signaling pathway and inhibits intestinal cell apoptosis via the TNF‐α/caspase‐8 signaling cascade, thereby reducing rectal injury.

Research on the pharmacological mechanisms of action of traditional Chinese medicine (TCM) has gradually deepened. Compared with chemical and biological drugs, TCM has unique advantages, such as fewer adverse effects and complex compositions involving multiple components with multiple targets. Ginseng is an herbal TCM that has been used for over 2000 years. It is an important TCM tonic with extensive medicinal value. GRg3, with the molecular formula C_42_H_72_O_13_, inhibits tumor angiogenesis,^11^ regulates the tumor cell cycle,^4^ and improves chemotherapeutic drug sensitivity.^12^ Several studies have revealed that GRg3 exerts potent anti‐inflammatory effects. It induces M2 macrophage polarization to accelerate inflammation regression and to inhibit mast cell‐mediated allergic inflammation via the MAPK/NF‐κB signaling pathway.^13^ In sepsis models, GRg3 exerts protective mitochondrial effects by activating the AMP‐activated protein kinase (AMPK) signaling pathway, which promotes mitochondrial autophagy. AMPK serves as a target for GRg3‐induced mitochondrial autophagy and alleviates oxidative stress, thereby protecting cells and organs from sepsis‐induced damage.^14^ As ARP is essentially an inflammatory response and GRg3 can exert anti‐inflammatory effects through multiple pathways, it is plausible that GRg3 would have a therapeutic effect on radiation enteritis.

The inflammatory process induced by ionizing radiation is the primary cellular defense mechanism and is accompanied by the production of multiple pro‐ and anti‐inflammatory factors. An imbalance in inflammation‐related cytokine levels after radiation can cause continuous damage to cells and tissues.^15^ TNF‐α is an inflammatory cytokine involved in the acute phase inflammatory response and is mainly produced by activated macrophages.^16^ TNF‐α levels are significantly increased in the serum of patients with RP^17^ and in the rectal tissues of RP rat models.^18^ IL‐4 and IL‐10, anti‐inflammatory cytokines, are secreted by Th2 cells and downregulate the inflammatory response by preventing Th1 cell proliferation, blocking pro‐inflammatory cytokine production, and reducing the function of antigen‐presenting cells.^19^ Hong et al.^20^ showed that TNF‐α levels were increased and IL‐4 and IL‐10 levels were decreased in the intestinal mucosa of RP model rats. Radiation disrupts the balance of pro‐ and anti‐inflammatory factors in the gut to trigger RP, consistent with our results using RP model rat serum. GRg3 was recently shown to have a good curative effect on arthritis, neuritis, pneumonia, nephritis, and other inflammatory diseases; however, the mechanism of its anti‐inflammatory effects has been unclear.^21^, ^22^, ^23^, ^24^ By establishing ARP rat models and GRg3 gavage treatment, we showed that GRg3 not only improved the general condition of RP rats, including their mood, appetite, and body weight, but also significantly reduced histopathological intestinal mucosal damage. The mechanism may be related to improved inflammation‐related cytokine balance, as we found that the serum pro‐inflammatory cytokine TNF‐α levels were decreased and those of anti‐inflammatory factors IL‐4 and IL‐10 were increased in rats in the medium‐ and high‐dose GRg3‐treatment groups. The IR group's weight increased during the late gavage stage, possibly because the ARP phase had passed and rats were recovering from the disease. This recovery indicates that ARP has self‐limiting properties. However, the weight of rats in the medium‐ and high‐dose GRg3‐treatment groups was still significantly higher than that in the IR group (*p* < 0.05), and the histopathological results and serum inflammatory indices also proved that GRg3 had significant therapeutic effects on ARP model rats.

Activation of NF‐κB is important in cellular inflammatory injury and immune responses. Under normal conditions, both NF‐κB and IκB exist in the cytoplasm in inactive forms. Therefore, selective inhibition of NF‐κB signaling pathway activation is a target for inflammatory disease treatment. Drugs that are widely used clinically to regulate NF‐κB activity include glucocorticoids, nonsteroidal anti‐inflammatory drugs, and certain anti‐rheumatic drugs. Glucocorticoids can increase NF‐κB retention in the cytoplasm by inducing transcription and synthesis of IκB‐α,^25^ sulfasalazine can directly inhibit nuclear translocation of NF‐κB,^26^ and high‐dose aspirin can inhibit IKK activity.^27^ GRg3 has been confirmed to play a therapeutic role in various diseases by regulating the NF‐κB signaling pathway. Ma et al.^28^ showed that GRg3 can reduce hypertrophic scar formation in rabbit ears by inhibiting p‐IκB activity. In human inflammatory airway epithelial cells and asthmatic airway epithelial tissues, GRg3 reduces inflammatory factor secretion by downregulating NF‐κB activity.^23^ Additionally, GRg3 can inhibit colon cancer cell migration and proliferation by inhibiting the activity of NF‐κB.^29^, ^30^ In this study, RT‐qPCR and western blot analysis were used to detect the changes in expression of NF‐κB pathway‐related factors in the rectal tissue of ARP rats. The results suggested that GRg3 may improve the balance of inflammatory factors by inhibiting the expression of IKK‐β, reducing the phosphorylation of IκB‐α, and increasing the retention of NF‐κB p50 in the cytoplasm. Thus, it reduces the inflammatory response and promotes the repair of intestinal tissue damage.

Recent studies have shown that GRg3 can inhibit apoptosis. Lee et al.^31^ found that GRg3 treatment downregulated apoptotic factors p53, BAX, and cleaved caspase‐3 in a Huntington's disease cell model. Hu et al.^32^ suggested that GRg3 exerts a protective effect against H_2_O_2_‐induced hippocampal neuronal injury in mice by downregulating the expression of caspase‐3. Apoptosis, a biological process essential for normal development and tissue homeostasis in the human body, is initiated and executed by cysteine proteases. Under different conditions, ionizing radiation induces cell death via different signal transduction mechanisms. However, as an initiator of apoptosis, activation of caspase‐8 is crucial^33^ and is an important factor in preventing radiation‐induced damage. Furthermore, GRg3 can significantly reduce the level of the apoptotic protein BAX.^7^ The present experiment verified that the mRNA and protein levels of caspase‐8 in rat rectal tissues were significantly reduced after GRg3 intervention, indicating that GRg3 can inhibit the apoptosis of intestinal cells induced by ionizing radiation. Additionally, TNF‐α levels were also significantly decreased in rats after GRg3 treatment. Thus, GRg3 may alleviate radiation‐induced rectal injury by inhibiting the TNF‐α/caspase‐8 signaling cascade and inhibiting intestinal cell apoptosis.

This study has some shortcomings. For example, the observation of the general condition of rats is subjective to some extent, and the judgment of rectal injury is only a qualitative analysis, which needs to be further quantified to improve the accuracy of the experiment. In addition, whether the RP application of GRg3 conflicts with its antitumor effect needs further experimental verification.

In conclusion, GRg3 can reduce rectal tissue damage in RP model rats through NF‐κB and TNF‐α/caspase‐8 signaling pathways, exerting anti‐inflammatory effects and inhibiting apoptosis, thereby reducing the severity of RP. Thus, GRg3 is a promising therapeutic agent for RP. In the future, the effect of GRg3 on the interaction between NF‐κB and caspase‐8 signaling pathways can be further elucidated by studying the molecular mechanism of the interaction.

## AUTHOR CONTRIBUTIONS

**Xuxia Li:** Conceptualization; data curation; formal analysis; investigation; methodology; project administration; software; supervision; validation; writing—original draft; writing—review and editing. **Lili Lin:** Conceptualization; formal analysis; investigation; methodology; project administration; software; writing—review and editing. **Xiaoyu Duan:** Investigation. **Jiuju Dai:** Validation. **Tingting Hu:** Validation. **Hongyi Cai:** Data curation.

## CONFLICT OF INTEREST STATEMENT

The authors declare no conflict of interest.

## ETHICS STATEMENT

This study and experimental procedures were approved by the ethics committee of Gansu Provincial People's Hospital. All animal housing and experiments were conducted in strict accordance with the institutional guidelines for care and use of laboratory animals. A completed ARRIVE guidelines checklist is included in supplementary materials (Checklist S1).

## Supporting information

Supporting information.

## Data Availability

The authors confirm that all data underlying the findings are fully available without restriction. All relevant data are within the paper and its Supporting Information files.
