# Correction to “Efficacy and Mechanism of Action of Ginsenoside Rg3 on Radiation Proctitis in Rats”

**DOI:** 10.1002/iid3.70086

**Published:** 2024-11-27

X. Li, L. Lin, X. Duan, J. Dai, T. Hu, and H. Cai, “Efficacy and Mechanism of Action of Ginsenoside Rg3 on Radiation Proctitis in Rats,” *Immunity, Inflammation and Disease* 12 (2024): e70015, https://doi.org/10.1002/iid3.70015.

In the article, the following errors were identified.

**Author Affiliation**

Lili Lin's affiliation was originally stated as ^2^Department of Oncology, Suqian First People's Hospital, Suqian, China. This should be ^2^Department of Oncology, Suqian First Hospital, Suqian China.

**Correspondence:**

Lili Lin was inadvertently missed from the original correspondence and should be added. The correspondence should read:

Lili Lin, Department of Oncology, Suqian First People's Hospital, Suqian, China. Email: linlilishijie@163.com

Hongyi Cai, Department of Radiotherapy, Gansu Provincial Hospital, 204 Donggang West Rd, Lanzhou, Gansu, China. Email: gschy163@163.com and 1143864940@qq.com

**Footnote:**

Xuxia Li and Lili Lin did not contribute equally to this paper and this footnote should be removed from the article.

The online version of the article has been corrected.

We apologize for these errors.

